# Deficits in force production during multifinger tasks demarcate cognitive dysfunction

**DOI:** 10.1007/s40520-024-02723-9

**Published:** 2024-04-05

**Authors:** Richard G. Carson, Debora Berdondini, Maebh Crosbie, Caoilan McConville, Shannon Forbes, Marla Stewart, Ruth Zhi Xian Chiu

**Affiliations:** 1 Trinity College Institute of Neuroscience and School of Psychology, Trinity College Dublin, Dublin 2, Ireland; 2 School of Psychology, Queen’s University Belfast, Belfast, Northern Ireland UK

**Keywords:** Cognition, Grip strength, Physical function, Coordination, Dexterity

## Abstract

**Background:**

The multifinger force deficit (MFFD) is the decline in force generated by each finger as the number of fingers contributing to an action is increased. It has been shown to associate with cognitive status.

**Aims:**

The aim was to establish whether a particularly challenging form of multifinger grip dynamometry, that provides minimal tactile feedback via cutaneous receptors and requires active compensation for reaction forces, will yield an MFFD that is more sensitive to cognitive status.

**Methods:**

Associations between measures of motor function, and cognitive status (Montreal Cognitive Assessment [MoCA]) and latent components of cognitive function (derived from 11 tests using principal component analysis), were estimated cross-sectionally using generalized partial rank correlations. The participants (*n*  =  62) were community dwelling, aged 65–87.

**Results:**

Approximately half the participants were unable to complete the dynamometry task successfully. Cognitive status demarcated individuals who could perform the task from those who could not. Among those who complied with the task requirements, the MFFD was negatively correlated with MoCA scores—those with the highest MoCA scores tended to exhibit the smallest deficits, and vice versa. There were corresponding associations with latent components of cognitive function.

**Discussion:**

The results support the view that neurodegenerative processes that are a feature of normal and pathological aging exert corresponding effects on expressions of motor coordination—in multifinger tasks, and cognitive sufficiency, due to their dependence on shared neural systems.

**Conclusions:**

The outcomes add weight to the assertion that deficits in force production during multifinger tasks are sensitive to cognitive dysfunction.

**Supplementary Information:**

The online version contains supplementary material available at 10.1007/s40520-024-02723-9.

## Introduction

Longitudinal studies indicate that in older adults, rates of decline in grip strength (GS) and cognitive function are closely aligned [1]. Although GS is often treated as a proxy for “muscular fitness” (e.g., [2]), there are no indications that cognitive and muscle function are linked [3, 4]. Since the effective application of grip force demands sophisticated neural control [5], it has been argued that a decline in GS which emerges beyond middle age should instead be considered a marker of brain health [6]. Accordingly, meta-analyses (27K–180K individuals) of prospective cohort studies [7, 8] and massive (> 250K individuals) population-based investigations [9] are consistent in demonstrating that individuals with low GS at baseline have higher (multivariable adjusted) risk of: cognitive impairment, cognitive decline, dementia, Alzheimer’s disease, and vascular dementia. In view of its power to anticipate impairment, it has been recommended that measurements of GS should be used to identify older adults at risk of neurological degeneration and cognitive decline [10–12].

The classifying power of GS in relation to cognitive function is independent of variations in muscle mass [13, 14]. Nonetheless, morphometric factors, including height, body-mass, and hand size, contribute to individual differences in GS (e.g., [15]). As they do so independently of variations in brain health that account for the associations between GS and cognition, differences in individual morphology necessarily reduce the sensitivity with which a conventional measurement of GS can provide prognosis of cognitive decline [16].

**Fig. 1:**
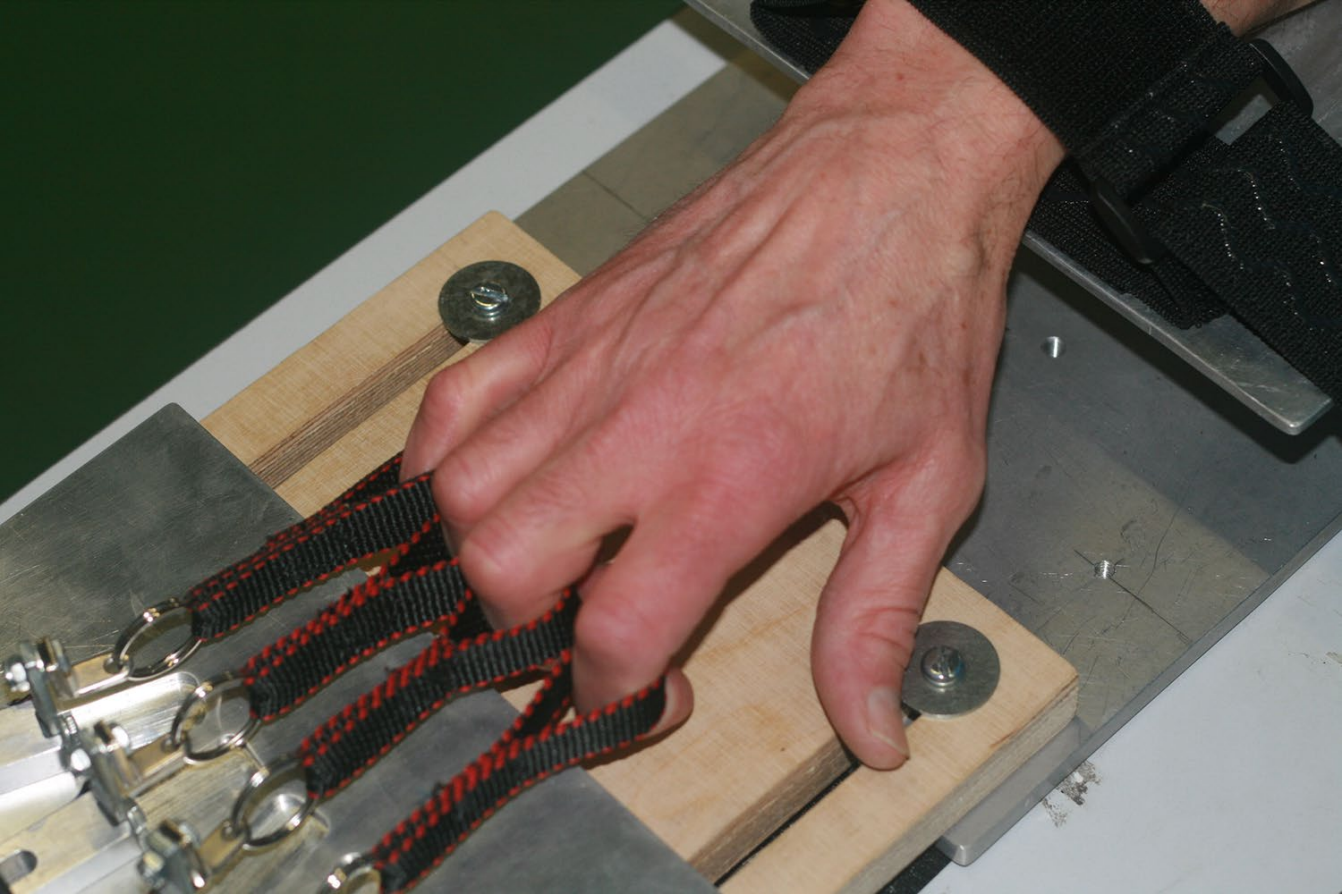
Multifinger (flexion) dynamometry implemented by recording the tension generated by flexion of the metacarpophalangeal and interphalangeal joints, via loops placed around the middle phalanges of digits I–IV. The loops are fixed—via a turnbuckle and cable assembly to (e.g., s-beam style) force sensors. The position of each turnbuckle and cable assembly can be adjusted for each individual to take up any slack in the coupling

With a suitably configured device, it is possible to register independently the force generated by each finger, either when used in isolation, or in combination with the other fingers (Fig. 1). The maximum force that can be produced by each finger decreases in proportion to the number of other fingers that are engaged simultaneously [17]. When all four fingers are engaged together, the force applied by each finger is typically about half that which can be applied when it is used in isolation. This is referred to as the “multi-finger force deficit” (MFFD). As the measure is relative, the absolute level of force that can be generated (by any finger) has no bearing on the magnitude of the deficit that is calculated. The MFFD thus provides a measure of neural sufficiency that is insensitive to the influence of morphometric factors that contribute to individual differences in conventional GS assessments. It is larger in older persons than in the young [18, 19], and in both the more impaired and less impaired limbs of those who have incurred brain damage following stroke [20].

We have shown [21] that the association between the MFFD, and cognitive status—measured with the Montreal Cognitive Assessment (MoCA), is greater than that which has been reported previously for GS [22]. Furthermore, the sensitivity with which the MFFD reflected cognitive decline was enhanced using as the basis for its calculation, the maximum rate of force (rof) developed by each finger, rather than the maximum force generated by each finger. Thus, expressions of the demands imposed upon the CNS with respect to muscle recruitment and coordination depend both on the characteristics of the task, and the facet of performance that is measured. The transducers used in our previous study to record the force applied by each finger were in the style of a “button” (covered with sandpaper) placed on a flat surface, on which downward pressure was applied. The MFFD has also been studied by attaching transducers to loops placed around each finger, and recording the tension generated by flexion of the metacarpophalangeal and interphalangeal joints [17]. To achieve the maximum possible forces, this variant (“flexion dynamometry”) requires that there is accurate control of the direction in which joint torque is applied, and attenuation of moments of force that would otherwise rotate the hand [23]. Tactile feedback, such as that provided when pressure is applied by a fingertip upon a button, plays a critical role in maximum finger force production. Augmentation of tactile feedback decreases [24], and digital anaesthesia increases [25] the magnitude of the MFFD. Tactile feedback is, however, diminished in flexion dynamometry, as the fingertips are not in contact with a surface. Being a task that places considerable demands on the CNS (with respect to muscle coordination), and which is impoverished with respect to tactile feedback, we hypothesised that flexion dynamometry will yield an MFFD that is particularly sensitive to cognitive status in older adults.

The MoCA was designed as a screening tool to detect individuals with mild cognitive impairment (MCI) [26]. Notionally, it encompasses several cognitive domains. One concern that arises from the use of the MoCA as a measure of cognitive status is that, as the domains (and the contributions of each component to the total score) are specified a priori, latent relationships (reflecting the multiple facets of cognition) with other CNS functions may be masked [27]. Here we used eight neuropsychological evaluations (yielding 11 separate scores) and principal component analysis (PCA) as a dimension reduction method to extract latent components.

## Materials and methods

### Participants

Based on the correlation of −0.38 between the rofMFFD and MoCA obtained by Carson and Holton [21]; *α*  = 0.05; power (1 − *β* error prob) = 0.80; a minimum sample size of 45 was estimated for the Kendall coefficient using the method of Looney [28]. Sixty-two volunteers took part in the study (42 females, 20 males, range 65–87, median 72.5 years) (see Supplementary Information). All provided written informed consent to procedures approved by Trinity College Dublin School (SPREC092021-28) and Queen’s University Belfast (EPS 21_330) ethics committees. Excepting preregistration, all testing was conducted in accordance with the Declaration of Helsinki. For all participants, the entire testing protocol was undertaken on a single day.

### Procedure

Data were anonymous at source. Each participant was assigned a randomly generated alphanumeric code, which was not linked to personal or identifying information. Age, height, and weight were recorded, along with years of formal education and qualifications achieved, followed by completion of the Edinburgh Handedness Inventory [29]. A standard assessment of grip strength (three attempts with the right hand) was undertaken using a Jamar Plus Digital Hand Dynamometer. Participants then performed the Timed Up and Go (TUG) [30], and the nine hole peg test [31].

Due to the physical configuration of the equipment used for multifinger dynamometry (see below), it was necessary for the right hand to be employed. To ensure consistency, the right hand was therefore also used for the standard assessment of grip strength—regardless of the handedness of the participant. Of the 62 persons tested, four were assessed to be left-hand dominant. In statistical analyses, a measure of laterality derived from the Edinburgh Handedness Inventory was included as a covariate.

### Multifinger dynamometry

Multifinger flexion dynamometry followed Ohtsuki [17]. The measurement device (Fig. 1) comprised fabric ‘finger rings’ looped around the middle phalanx of each finger. Each was coupled to a force transducer via a turnbuckle and cable assembly. The participant placed their right forearm in a prone position on a supporting platform, with the elbow in approximately 90 degrees of flexion. Webbing straps were used to maintain constant the position and posture of the forearm.

A data collection sequence consisted of ten applications of force—two for each finger separately, and two when applying force with all four fingers simultaneously. The order of these conditions within the sequence was randomised. Prior to each application of force, a computer-synthesised voice specified the finger/fingers to be used: “index finger”; “middle finger”; “ring finger”; “pinkie finger”; or “all fingers” (“pinkie finger” was used, as “little finger” sounds similar to “middle finger”). The participant was then prompted to start applying force—at maximum effort, by an auditory tone (400 Hz; 1 s duration). Once 3 s had elapsed, a synthesised positive feedback sound indicated that the participant should cease applying force. The interval between successive applications of force was approximately 10 s. Six such sequences were undertaken. The interval between successive sequences was 2 min. Thus, there were 12 applications of force when each finger was used in isolation, and 12 applications of force when all four fingers were to be used together. To familiarise the participants with the task, one block of ten practice trials was first undertaken [32]. Only the right hand was assessed. The transducer signals were amplified and digitised at 200 Hz with 16-bit resolution (National Instrument, BNC-2090A). The control of data acquisition and cue presentation was implemented via custom MATLAB routines. The duration of this first phase of testing was approximately 45 min.

### Cognitive testing

There was a break of 10 min before the tests of cognitive function. The 12th November 2004 version of the MoCA was administered. This was followed by eight tests selected to provide broad coverage of cognitive functions (see Supplementary Information). These were the Victoria Stroop Test; the Digit Span Task (forward and backward); the Trail Making Test (A and B); the Weigl Colour‐Form Sorting Test; the Mental Rotation Task; the Controlled Oral Word Association Test (COWAT); the short version of the Numerical Activities of Daily Living (NADL) test; and the Continuous Card Sorting Task—which is a Choice Reaction Time (CRT) variant.

### Data processing

Level of education is one of the variables considered in the fully adjusted norms for the MoCA [26]. This was scored (range 0–8) following the ISCED 2011 Operational Manual Guidelines for classifying national education programmes and related qualifications [33].

The measure obtained for the Victoria Stroop Test was the difference between the times taken to complete the Neutral Word and (incongruent) Colour Word variants. For the Trail Making Test, it was the difference between the times taken to complete the A and B variants. In the language of information theory, the information content for the four variants of the CRT was: 0 bits; 1 bit, 2 bits; and 4.7 bits. The derived measure for each participant was taken as the slope of a linear function relating sort time to information content. The values obtained for the forward and backward version of the Digit Span Task were treated as separate variables. The scores obtained for the word and animal variants of the COWAT were treated as separate variables. For each participant, therefore, 11 cognitive test scores were included in the PCA.

Robust PCA (see Supplementary Information) was implemented in R [34], using the pca() function from the pcaMethods package [35]. Prior to undertaking the PCA, all values (separately for each test) were scaled (normalised with respect to the median absolute deviation) and centred (with respect to the median). Successive PCs provide diminishing returns, in terms of the degree to which they account for variance present in the original data. The technique used to determine the number of components that should be retained for further analysis, was that proposed by Minka [36], whereby the relevant number is that which maximizes the posterior Bayesian information criterion (BIC).

Analysis of the force recordings was undertaken by a member of the research team who was not aware of the scores achieved by the participants on any of the tests of cognitive function. Force time series were low-pass filtered digitally at 6 Hz with a second-order, dual-pass Butterworth filter. Differentiation to obtain the rate of force (rof) application was by estimation of the slopes of five-point first-order polynomials fitted to the force time series. For each trial, the maximum force applied by each finger (N), and the maximum rate of force (Ns^−1^) was identified.

All finger force recordings were inspected visually with the aim of identifying (1) trials on which the finger(s) engaged by the participant were not in accordance with the instruction given, and (2) trials on which the participant commenced the application of force in advance of the signal to do so (in such instances the maximum rate of force could not be determined accurately). Any such trials were excluded from analysis. An algorithm was then applied (without human intervention) to identify and exclude trials on which the maximum force value corresponded to the final sample of the 3 s recording interval (indicating that the force was still increasing).

The magnitude of the MFFD was calculated by first expressing for each finger the (mean) maximum force generated in the multifinger condition, as a ratio of the (mean) maximum force generated when that finger was used in isolation. It was a requirement that a minimum of six trials were available for each condition. The mean of the four ratios thus generated (i.e., one for each finger) was subtracted from 1 to obtain the MFFD for the participant. Corresponding calculations were undertaken to obtain the rofMFFD. This was the (mean) maximum rof generated in the multifinger condition, as a ratio of the (mean) maximum rof generated when that finger was used in isolation [21].

### Statistical analysis

The MFFD and rofMFFD are ratio measures, which have disagreeable statistical properties (e.g., [37]). Similar concerns extend to the MoCA, due to the truncated range within which the scores lie, and to eigenvalue-based methods such as PCA, which can generate non-normal, non-symmetric distributions of component scores [38]. Robust statistical analysis methods were therefore used. Following the approach described in Carson and Holton [21], generalized partial rank correlations [39], implemented in R via the taba package, were used to determine the degree of monotonic association between (separately) the MoCA and PCA-derived estimates of cognitive status and, respectively, the MFFD and the rofMFFD. With respect to the measures of cognition, age and ISCED classification were included as covariates. For the MFFD and rofMFFD, the covariates were age and the Laterality Quotient (LQ: from the Edinburgh Handedness Inventory). As the motivating hypotheses specified negative relationships between cognitive status and the MFFD and rofMFFD, respectively, one-tailed tests were indicated. Since the associations with PCA-derived estimates of cognitive status were exploratory, Benjamini–Hochberg false discovery rates (FDR) [40] were calculated. An attractive feature of this approach is that an FDR estimate can be given a Bayesian interpretation, and viewed as the propensity for a claim that an association is present, to be false [41, 42]. As percentile bootstrap methods are not noticeably superior for samples smaller than 200, and correlations that exceed 0.5 [43], confidence intervals for the generalized partial correlation coefficients were calculated using a Fisher transformation.

## Results

The flexion dynamometry task proved to be challenging for many people. Indeed, only 30 of the 62 participants satisfied the requirement that a minimum of six valid trials be completed in every condition. In most such cases, there was a failure to register a maximum force value for all fingers by the end of the (3 s) recording interval, or initiation of force application in advance of the signal to do so. These features were most frequently exhibited in the multifinger conditions. We therefore sought to determine whether the capacity to execute the task was associated with the cognitive status of the participants. A robust logistic regression [using the rlm() function from the MASS package], which included age as a covariate, indicated that MoCA score was reliably associated with flexion dynamometry task completion (Estimate = 0.03, *p* = 0.044 (one-tailed), 0.001–0.068 [95% CI]). No such association was apparent for age (estimate = 0.004, *p* = 0.387 (one-tailed), −0.018 to 0.024 [95% CI]).

The range of MoCA scores for the entire sample was 15–30 (median = 26). With respect to the 30 participants who satisfied the task requirements, the range of MoCA scores was 17–30 (median = 27). Among those who failed to satisfy these requirements, the range of MoCA scores was 15–30 (median = 25.5). Necessarily, measures of association for the MFFD and rofMFFD were based only on participants who satisfied the task requirements.

**Fig. 2:**
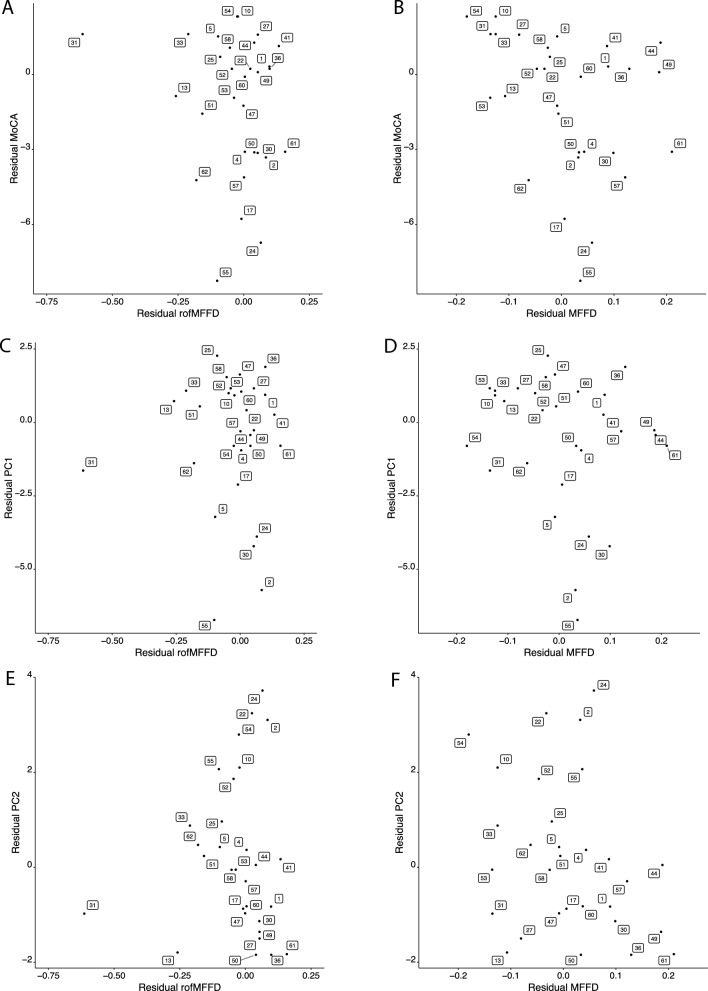
It is generally recommended that, in respect of the scatter of partial correlations, partial regression plots (residuals of Y on the corresponding covariates versus residuals of X on the corresponding covariates) be used [66]. For these illustrative purposes, a rank-based estimation model (implemented using the Rfit package in R [67] was used to generate the residuals. **A** The residuals for the MoCA (i.e., which partial out the variation attributable to age and ISCED) plotted with respect to the residuals for the rofMFFD (i.e., which partial out the variation attributable to age and LQ). **B** The residuals for the MoCA (i.e., which partial out the variation attributable to age and ISCED) plotted with respect to the residuals for the MFFD (i.e., which partial out the variation attributable to age and LQ). **C** The residuals for the PC1 scores (i.e., which partial out the variation attributable to age and ISCED) plotted with respect to the residuals for the rofMFFD (i.e., which partial out the variation attributable to age and LQ). **D** The residuals for the for the PC1 scores plotted with respect to the residuals for the MFFD (i.e., which partial out the variation attributable to age and LQ). E. The residuals for the PC1 scores (i.e., which partial out the variation attributable to age and ISCED) plotted with respect to the residuals for the rofMFFD (i.e., which partial out the variation attributable to age and LQ). F. The residuals for the for the PC1 scores plotted with respect to the residuals for the MFFD (i.e., which partial out the variation attributable to age and LQ). In all panels, the labels shown for individual datapoints correspond to the participant identifiers (IDs) given in Table 1

Replicating the observation reported by Carson and Holton [21], the magnitude of the rofMFFD was associated with the MoCA score. The generalized partial rank correlation between the rofMFFD and the MoCA was −0.47 (*p* = 0.007 [one-tailed], −1 to −0.19 [95% CI], *n* = 30). That between the MFFD and the MoCA was −0.51 (*p* = 0.003 [one-tailed], −1 to 0.24 (95% CI), *n* = 30). The two MFFD variants were not distinguished clearly in terms of the magnitude of the association with the MoCA (Fig. 2A, B).

**Fig. 3:**
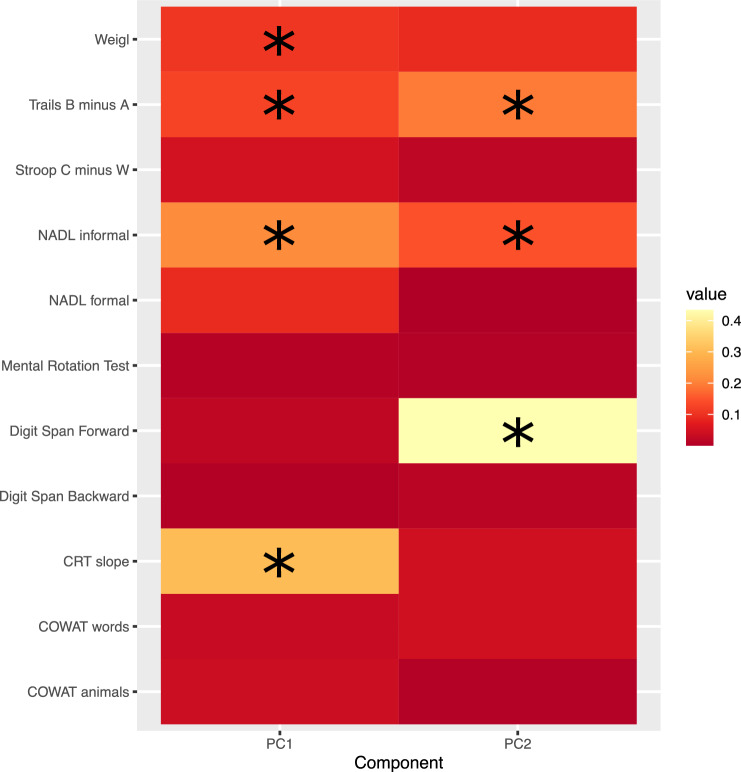
A heatmap visualization of the PCA solutions for the tests of cognitive function. The colour saturation corresponds to the squares of the loadings for each test onto PC1 and PC2, respectively. The asterisk (*) symbol indicates tests for which the squared magnitude of the loading onto the PC exceeds 0.1

Retention of only the first two PCs was indicated. The loadings of the individual cognitive test scores onto the components are given in Table 1. The squares of the loadings are represented in Fig. 3. These values can be interpreted as the proportion of the variance in each PC accounted for by the respective test scores.

**Table 1:** Loadings of the cognitive test outcome measures onto the principal components

	PC1	PC2
Digit span forward	0.148	0.657
Digit span backward	0.068	0.128
Weigl test	0.327	0.300
Mental rotation tests	0.089	0.074
COWAT words	0.178	0.209
COWAT animals	0.196	0.075
NADL informal	0.466	−0.383
NADL formal	0.299	−0.038
Stroop colours—neutral	−0.220	0.144
Trails B–Trails A	−0.356	0.441
CCST slope	−0.554	−0.210

Through this process, a value was generated for each participant for each PC. These values were analyses in the fashion described for the MoCA. The generalized partial rank correlation between the rofMFFD and PC1 was −0.19 (−1 to 0.12 [95% CI] (one-tailed), FDR = 0.169, *n* = 30). That between the MFFD and PC1 was −0.41 (−1 to 0.12 (95% CI) (one-tailed), FDR = 0.021, *n* = 30) (Fig. 2C, D). The generalized partial rank correlation between the rofMFFD and PC2 was −0.65 (−1 to −0.43 (95% CI) (one-tailed), FDR = 0.0003, *n* = 30). That between the MFFD and PC2 was also −0.65 (−1 to −0.43 [95% CI] [one-tailed], FDR = 0.0005, *n* = 30) (Fig. 2E, F). The FDR propensity that the finding of an association between MFFD and PC1 is mistaken was estimated to be approximately 2%. The FDR propensity that the findings of associations between the MFFD and rofMFFD, respectively, and PC2 were erroneous was less than 0.001% in both cases.

### Subsidiary analyses

The degree of monotonic association between (separately) the MoCA and (1) a conventional measure of GS, obtained as the maximum force generated in three attempts; (2) the time taken to complete the 9-hole peg test; (3) the time recorded for the TUG, was determined. In respect of GS, the height and weight of the participant was included as a covariate. For the 9-hole peg test, the model described for the MFFD and rofMFFD was used. The height and weight of the participant were included as covariates in the model for TUG time. The results are reported separately for the 30 participants who met the requirements of the flexion dynamometry task (Table 2), and for the 32 participants who did not (Table 3).

**Table 2:** Participants who met the requirements of the flexion dynamometry task (*n* = 30)

Association	Coefficient	Lower 95% CI	Upper 95% CI	FDR
MoCA with GS	0.54	0.28	1	0.016
MoCA with pegboard	−0.45	−1	−0.16	0.020
MoCA with TUG	0.06	−1	0.36	0.47

Summary of generalized partial rank correlations representing the magnitude of the monotonic association between the MoCA and (1) a conventional measure of grip strength (GS); (2) the time taken to complete the 9-hole peg test; (3) the time recorded for completion of the timed up and go (TUG). For each correlation coefficient, the corresponding lower and upper 95% confidence interval (CI) is given, in accordance with the use of directional hypothesis and one-tailed test. The false discovery rate (FDR) value may be interpreted in a Bayesian context as the propensity that a non-null claim is, in fact, wrong

**Table 3:** Participants who failed to meet the requirements of the flexion dynamometry task (*n* = 30)

Association	Coefficient	Lower 95% CI	Upper 95% CI	FDR
MoCA with GS	0.49	0.23	1	0.014
MoCA with pegboard	−0.34	−1	−0.05	0.052
MoCA with TUG	0.00	−1	0.30	0.50

Summary of generalized partial rank correlations representing the magnitude of the monotonic association between the MoCA and (1) a conventional measure of grip strength (GS); (2) the time taken to complete the 9-hole peg test; (3) the time recorded for completion of the timed up and go (TUG). For each correlation coefficient, the corresponding lower and upper 95% confidence interval (CI) is given, in accordance with the use of directional hypothesis and one-tailed test. The false discovery rate (FDR) value may be interpreted in a Bayesian context as the propensity that a non-null claim is, in fact, wrong

There was little to suggest the presence of statistical associations between the TUG time and MoCA scores. For both samples, there was a negative association between the times to complete the 9-hole peg test and MoCA scores. That is, there was a propensity for those with higher MoCA scores to take less time to complete the peg test, and for those with lower MoCA scores to take more time to complete the peg test. Maximum GS was positively associated with MoCA scores. Individuals with lower MoCA scores tended to have lower GS, and individuals with higher MoCA scores tended to have greater GS.

## Discussion

The hypothesis that motivated the study is that the MFFD is a measure of CNS sufficiency, which can serve as a proxy measure of functional brain integrity and provide a marker of cognitive impairment [16]. The MFFD has been computed using several forms of dynamometry. In some, it is necessary that the moments at individual fingers produced by reaction forces are counterbalanced by the action of muscles both within the hand, and those which cross the wrist and radioulnar joint. If there is no secondary moment at the wrist and radioulnar joint, the magnitude of the MFFD is diminished [44]. In other forms of dynamometry, such as those in which the fingertips apply pressure on buttons, cutaneous receptors in the pads of the distal phalanges provide tactile feedback. This also reduces the magnitude of the MFFD [25]. In the present investigation, we used a form of dynamometry that required active compensation for secondary moments produced by reaction forces and did not stimulate cutaneous receptors in the pads of the fingers. We reasoned that these factors would accentuate the demands placed on the CNS and provide a particularly sensitive measure of functional brain integrity and cognitive impairment.

Our expectation was realised in an unexpected fashion. Approximately half of the participants were unable to complete the task in accordance with requirements. In many cases, an asymptotic value of the applied force was not achieved by all fingers during the 3 s data recording interval. In others, force was produced before it was necessary. In short, this form of dynamometry was just too difficult for some people. With this realisation, we considered the possibility that the individual capability to perform the task may have been related to cognitive status. We used logistic regression to model the probability of successful task completion, with MoCA test score and age as independent variables. This analysis indicated that the probability of task completion was associated with cognitive status—those with higher MoCA scores were more likely to satisfy the requirements of the task. There was no association with age. Although the median MoCA score for the individuals who were unable to perform the task (25.5) was only 1.5 points lower than that of the group that were able to do so, a value below 26 accords with a designation of “mild cognitive impairment” (MCI) in the scheme proposed originally by Nasreddine et al. [26].

Often unseen, all actions require anticipatory postural adjustments (APAs), that compensate in advance for the disturbances to equilibrium brought about by voluntary movement [45]. The formulation of muscle synergies that support the coordinated application of grip force depends critically on CNS mechanisms that implement such forward planning [6]. In the flexion dynamometry task described here, the synchronous application of force by all four fingers required anticipatory compensation for the moments produced by reaction forces. Being anticipatory, the timing of such compensation is critical. It precipitates the focal action. The failure on the part of some participants to achieve asymptotic levels of force for all four fingers within the time allocated suggests belated anticipatory compensation. In this vein, individuals with a designation of MCI exhibit APAs (during gait) that are delayed relative to those with MoCA score ≥ 27 [46]. On the other hand, the advance initiation of force application also observed in the present study may indicate that, in expectation of the challenge of completing the task in the time allocated, some participants acted pre-emptively. In this regard, during arm-pointing movements, APAs that are advanced in time relative to those of age-matched controls have been reported for individuals with MCI [47]. The more general inference is that the anticipatory motor control required by grip dynamometry draws on CNS resources that extend beyond the classical motor network, to encompass other regions that play a role in cognition [48, 49]. The significant requirement for anticipatory control imposed by the multifinger flexion dynamometry used in the current investigation may therefore account for the association between task competency and cognitive status.

Necessarily, statistical associations between cognitive status and the magnitude of the MFFD and rofMFFD, respectively, were derived only for participants who were able to complete the task. There were negative associations in both cases. That is, those individuals with the highest scores on the MoCA tended to exhibit the smallest deficits, and those with lower MoCA scores tended to exhibit the largest deficits. Carson and Holton [21] reported that the association with MoCA scores was greater for the rofMFFD than for the MFFD, when these were derived using button press dynamometry. In the present case, the magnitudes of the associations (−0.47 and −0.51, respectively) could not be distinguished with confidence. Comparable values for correlations between tests of motor function and screening tools akin to the MoCA (e.g., the Mini-Mental State Examination [MMSE]) have only rarely been reported (e.g., [50, 51]). In most cases, the reported associations are very much weaker (< 0.20, e.g., [52, 53]).

In the present study, we sought to extend upon approaches in which cognitive status is represented by a compound score (such as the MoCA), for which the contributions of particular “domains” are specified a priori (e.g., [27]). This was accomplished using PCA to extract the latent components constituted by a set of individual predictors (i.e., cognitive tests), and assigning to each participant a score for each relevant component. There are several advantages to this approach. Component scores capitalise upon statistical aggregation to reduce error variance [54], they can compensate for ceiling or floor effects that may be associated with individual measures [55], and tend to be more reliable than those of the individual tests [56, 57]. They are particularly appropriate when samples consist of individuals who vary markedly in respect of cognitive status [58]. We opted to use PCA, rather than exploratory factor analysis (EFA), as in PCA, the linear combinations maximize the total variance, whereas in EFA, the linear combinations maximize the shared portion of the variance. Furthermore, PCA entails no prior assumption concerning the model (such as that correlation among the manifest variables is due to one or more common latent factors).

It was determined empirically that two PCs were sufficient to encapsulate the variance present in the 11 original test scores. With respect to the first PC, the largest loadings were obtained for: the slope across levels of the CRT task; the NADL informal; the Trails B–Trails A; and the Weigl test (in each case accounting for more than 10% of the total variance). With respect to the second PC, the largest loadings were obtained for: Digit Span Forward; Trails B–Trails A; and the NADL informal. While it may be tempting to map such loadings patterns onto terms such as “executive function” or “working memory”, these are psychological constructs lacking clear relations to specific anatomical structures or physiological processes [59, 60]. More generally, it is inadvisable to presume that the brain ‘divides up its functions into categories which correspond to our concepts or vocabulary’ [61]. We therefore do not go beyond emphasizing here that there were particularly high degrees of association between the scores for PC2 and the rofMFFD and MFFD, respectively (both correlation coefficients = −0.65). The MFFD was also associated (correlation = −0.41) with the PC1 scores. The FDR propensity that this finding is mistaken, was estimated at approximately 2%. In contrast, the association between the rofMFFD and the PC1 scores (−0.19) is less likely to be trustworthy (FDR propensity = 17%). In summary, for the MFFD, and to a somewhat lesser extent the rofMFFD (at least among those individuals who could successfully perform the flexion dynamometry task), there were clear associations both with a commonly used measure of cognitive status (i.e., the MoCA), and with scores reflecting the latent components constituted by a set of 11 individual predictors (generated using eight tests selected to provide broad coverage of cognitive functions). We consider these results to be consistent with the more general assertion that changes in central neurological processes are a common factor mediating the close relationship that exists between lifespan variations in motor coordination and cognition [6]. As they depend, at least in part, upon shared neural systems, it is to be anticipated that the neurodegenerative processes that are a feature of normal and pathological aging will exert corresponding effects on expressions of motor coordination and cognitive sufficiency.

It has variously been suggested that performance on other motor tests, such as the pegboard, TUG, and measurements of GS, can be related to incipient cognitive decline (e.g., [62]). Although in this study we were not able to monitor longitudinal changes, in subsidiary analyses, we sought to determine whether scores on these tests associated with cognitive status in the manner of the MFFD and rofMFFD. These analyses were undertaken separately for the group of participants that could successfully perform the flexion dynamometry task (Table 2), and for the group that could not (Table 3). The (sample-size weighted) mean magnitude of the correlation coefficient (0.52) obtained for GS and the MoCA, exceeded comparable meta-analytic estimates (largely based on the MMSE) obtained at baseline in longitudinal studies (*β* = 0.14, 95% CI 0.09–0.19) [52]. Corresponding meta-analytic estimates have not been generated for pegboard tests. In the only large ( > 1000) cross-sectional study of which we are aware, the correlation coefficient reported for an association with global cognition (MMSE) was −0.25 [63]. This is lower than the (sample-size weighted) mean value of −0.39 that we obtained (i.e., for the MoCA). It has been noted previously that that inter-individual variations in maximum GS are closely associated with those in manual dexterity. Furthermore, the strength of this association increases with age [64]. Larger samples and alternative inferential analysis models will, however, be required to determine whether there is a source of common variation in GS and manual dexterity, that accounts for the associations with cognitive status obtained for these measures. In this vein, it is notable that for the group that could successfully perform the flexion dynamometry task, the magnitudes of the associations with the MoCA (i.e., for the 9-hole pegboard and maximum GS) were not distinguishable from those obtained for the MFFD and rofMFFD (as indicated by the overlap of the respective confidence intervals).

## Limitations

The present study had several limitations. It was not a prospective study. Thus, it was not possible to test the hypothesis that the MFFD can be used to detect incipient cognitive decline [16]. Even though it was powered adequately in relation to the effect sizes reported by Carson and Holton [21], in absolute terms, the sample was small. As a result of our commitment to “paper and pencil” versions, the combination of tests used to assess cognitive function was unique. Even when different combinations of tests are used however, if data reduction methods such as PCA are applied, scores derived for latent variables correlate highly across test batteries [65].

As testing was undertaken in a single session of approximately two hours duration, it is possible that central fatigue—originating in the CNS and affecting cognitive and motor function, may have been induced. To the extent that any such fatigue (if resulting in a decrease in neural drive to spinal motoneurons or a change in the coordination of neural drive to spinal motoneurons) was expressed more prominently in the multifinger condition than in the single finger conditions, it may have had an impact on the magnitude of the MFFD that was registered. Similarly, central fatigue may influence performance on tests of cognitive function. Should the effects of central fatigue on the MFFD and on tests of cognitive function be coupled, and if individuals differ systematically in their susceptibility to such fatigue, some of the statistical associations observed may be accounted for in these terms. The presence of any such coupling would, however, suggest that the magnitude of the MFFD is not only a marker of systemic brain health [16], but also that it is sensitive to acute challenges, such as those posed by central fatigue.

## Conclusions

A form of dynamometry that required active compensation for secondary moments produced by reaction forces and which did not stimulate cutaneous receptors in the pads of the fingers was too difficult for some older adults. Individuals who could perform the task adequately were demarcated from those who could not, based on cognitive status (assessed using the MoCA). Among individuals who were able to complete the task, the decline in the force (and rate of force) generated when all four fingers were engaged simultaneously, relative to when each finger was used in isolation (the MFFD and rofMFFD), was negatively associated with cognitive status. Corresponding associations with latent components of cognitive function were also obtained. These results add further weight to the assertion [16] that deficits in force production during multifinger tasks reflect cognitive dysfunction.

### Supplementary Information

Below is the link to the electronic supplementary material.

Supplementary file1 (DOCX 22 KB)

## Data Availability

The datasets generated during and/analysed during the current study are available from the corresponding author on reasonable request.
