# Supplementary material for: Deficits in force production during multifinger tasks demarcate cognitive dysfunction
Source: Aging Clin Exp Res. 2024 Apr 5;36(1):87. doi: 10.1007/s40520-024-02723-9 (PMC10997684; doi:10.1007/s40520-024-02723-9)
Supplement: Supplementary file 1 — Supplementary file1 (DOCX 22 KB) [file 40520_2024_2723_MOESM1_ESM.docx]

**Supplementary Information**

*Additional Inclusion and Exclusion Criteria*

Non-probability convenience sampling was utilised to recruit participants who were community dwelling, aged 65 or older, healthy by self-declaration, spoke English as a first language and fully vaccinated for COVID-19. We did not seek to exclude individuals with discernible cognitive impairment. Advertising posters and cards were distributed on noticeboards in churches, pharmacies, sports centres, golf clubs, community groups and social clubs. In Belfast, Age NI, Engage with Age, the Open Learning Programme at Queen’s University Belfast, and the University of the Third Age also referred prospective participants to the research team. Snowball (chain-referral) sampling was implemented, whereby individuals who had completed the study, mentioned it to partners, friends, or family members. Negative responses to the following questions were required for enrolment:

Do you have difficulty understanding conversations because of your hearing even if you wear a hearing aid?

1. Do you have trouble with your vision that prevents you from reading ordinary print even when you have glasses on?
2. Do you have difficulty using your hands?
3. Do you require assistance when walking or using stairs?
4. Do you have any form of colour blindness?

*Tests of Cognitive Function*

Older people tend to have less experience with digital technologies than younger people, and may require additional time and assistance before feeling comfortable with computer-based cognitive tests (Fredrickson et al., 2010). Attitudes towards computers have a significant effect on cognitive test performance (Fazeli et al., 2013), and people who use computers frequently perform better on computer-based tests that require keyboard use or rapid visual scanning, and emphasise response time (Iverson et al., 2009). We therefore used standard (“paper and pencil”) versions of the cognitive tests.

The Victoria Stroop Test has good psychometric properties, including excellent test-retest reliability (coefficients of .90, .83, and .91 for time to complete the Dot, Neutral Word, and Color Word tasks, respectively) (Troyer et al., 2006). It has been established the Digit span task has acceptable psychometric properties when used with older adults (e.g., de Paula et al., 2016; Waters and Caplan, 2003). The internal reliability of the composite scores obtained for the Trail Making Test (A and B) exceeds 0.9, and test-retest reliability coefficients exceed 0.70 (Reynolds, 2002). The Weigl Colour‐Form Sorting Test (e.g., Laiacona et al., 2000) exhibits sensitivity and specificity of 83.3% and 94.1% respectively in healthy older adults (Hobson et al., 2007). In respect of the Mental Rotation Task (Peters et al., 1995), Cronbach's Alpha estimated by odd-even reliability has been reported as 0.91 (Voyer et al., 2006). In the present study, the MRT-A form was used. The Controlled Oral Word Association Test (Fogel, 1962) exhibits good internal consistency (Cronbach's alpha of 0.83) and test-retest reliability (R = .74) (Ruff et al., 1996). The whole formal test component of the NADL test has a Cronbach's alpha of 0.73 (Semenza et al., 2014). The short version correlates highly with the original battery (Kendall’s tau greater than 0.6 across tasks) and the cut-offs correctly identify impaired performance (accuracy of 95% or above) (Burgio et al., 2022). The continuous card sorting task (Crossman, 1953) was used as a convenient analogue of the choice reaction time task – which is typically automated. There have been no published determinations of the psychometric properties of this test.

*PCA Algorithm*

The sampling distributions of many cognitive tests are skewed (positively or negatively). In addition, it is not uncommon for outliers to be present. Classical PCA algorithms are designed to minimize the mean-square representation error or maximize the variances under orthonormality constraints. Both methods provide poor estimates in the presence of outliers or when the data are (positively) skewed (Karhunen, 2011). In recognition of this limitation, several robust PCA techniques have been developed (see Bouwmans and Zahzah, 2014 for review). For the present purposes, the “robustPca” argument to the pca() function from the pcaMethods package (Stacklies et al., 2007) was used. In this implementation, a robust approximation to the singular value decomposition of a rectangular matrix is computed using an alternating L1 (absolute value) norm minimization, rather than the conventional L2 (least squares) norm (Hawkins et al., 2002).

**References**

Bouwmans, T., Zahzah, E.H., 2014. Robust PCA via principal component pursuit: A review for a comparative evaluation in video surveillance. Chemometrics and Intelligent Laboratory Systems 122, 22–34. doi:10.1016/j.cviu.2013.11.009

Burgio, F., Danesin, L., Benavides-Varela, S., Meneghello, F., Butterworth, B., Arcara, G., Semenza, C., 2022. Numerical activities of daily living: a short version. Neurol. Sci. 43, 967–978. doi:10.1007/s10072-021-05391-z

Crossman, E.R.F.W., 1953. Entropy and choice time: The effect of frequency unbalance on choice-response. Quarterly Journal of Experimental Psycholohy 5, 41–51. doi:10.1080/17470215308416625

de Paula, J.J., Malloy-Diniz, L.F., Romano-Silva, M.A., 2016. Reliability of working memory assessment in neurocognitive disorders: a study of the Digit Span and Corsi Block-Tapping tasks. Braz J Psychiatry 38, 262–263. doi:10.1590/1516-4446-2015-1879

Fazeli, P.L., Ross, L.A., Vance, D.E., Ball, K., 2013. The relationship between computer experience and computerized cognitive test performance among older adults. J Gerontol B Psychol Sci Soc Sci 68, 337–346. doi:10.1093/geronb/gbs071

Fogel, M.L., 1962. The Gerstmann syndrome and the parietal symptom-complex. The Psychological Record 12, 85–99.

Fredrickson, J., Maruff, P., Woodward, M., Moore, L., Fredrickson, A., Sach, J., Darby, D., 2010. Evaluation of the usability of a brief computerized cognitive screening test in older people for epidemiological studies. Neuroepidemiology 34, 65–75. doi:10.1159/000264823

Hawkins, D.M., Liu, L., Young, S.S., 2002. Robust Singular Value Decomposition (No. Technical Report Number 122), National Institute of Statistical Sciences. Research Triangle Park, NC.

Hobson, P., Meara, J., Taylor, C., 2007. The Weigl Colour-Form Sorting Test: a quick and easily administered bedside screen for dementia and executive dysfunction. Int J Geriatr Psychiatry 22, 909–915. doi:10.1002/gps.1765

Iverson, G.L., Brooks, B.L., Ashton, V.L., Johnson, L.G., Gualtieri, C.T., 2009. Does familiarity with computers affect computerized neuropsychological test performance? J Clin Exp Neuropsychol 31, 594–604. doi:10.1080/13803390802372125

Karhunen, J., 2011. Robust PCA methods for complete and missing data. Asian Journal of Applied Sciences 21, 357.

Laiacona, M., Inzaghi, M.G., De Tanti, A., Capitani, E., 2000. Wisconsin card sorting test: a new global score, with Italian norms, and its relationship with the Weigl sorting test. Neurol. Sci. 21, 279–291. doi:10.1007/s100720070065

Peters, M., Laeng, B., Latham, K., Jackson, M., Zaiyouna, R., Richardson, C., 1995. A redrawn Vandenberg and Kuse mental rotations test: different versions and factors that affect performance. Brain and Cognition 28, 39–58. doi:10.1006/brcg.1995.1032

Reynolds, C.R., (null), 2002. Comprehensive trail making test. Pro-Ed 88–91. doi:DOI: 10.1177/0734282905282415

Ruff, R.M., Light, R.H., Parker, S.B., Levin, H.S., 1996. Benton Controlled Oral Word Association Test: reliability and updated norms. Arch Clin Neuropsychol 11, 329–338.

Semenza, C., Meneghello, F., Arcara, G., Burgio, F., Gnoato, F., Facchini, S., Benavides-Varela, S., Clementi, M., Butterworth, B., 2014. A new clinical tool for assessing numerical abilities in neurological diseases: numerical activities of daily living. Front Aging Neurosci 6, 112. doi:10.3389/fnagi.2014.00112

Stacklies, W., Redestig, H., Scholz, M., Walther, D., Selbig, J., 2007. pcaMethods--a bioconductor package providing PCA methods for incomplete data. Bioinformatics 23, 1164–1167. doi:10.1093/bioinformatics/btm069

Troyer, A.K., Leach, L., Strauss, E., 2006. Aging and response inhibition: Normative data for the Victoria Stroop Test. Neuropsychol Dev Cogn B Aging Neuropsychol Cogn 13, 20–35. doi:10.1080/138255890968187

Voyer, D., Butler, T., Cordero, J., Brake, B., Silbersweig, D., Stern, E., Imperato-McGinley, J., 2006. The relation between computerized and paper-and-pencil mental rotation tasks: a validation study. J Clin Exp Neuropsychol 28, 928–939. doi:10.1080/13803390591004310

Waters, G.S., Caplan, D., 2003. The reliability and stability of verbal working memory measures. Behav Res Methods Instrum Comput 35, 550–564. doi:10.3758/bf03195534
